# Structural promiscuity in the human circulatory IgA1 clonal repertoire

**DOI:** 10.1073/pnas.2533440123

**Published:** 2026-05-06

**Authors:** Amber D. Rolland, Sofia Kalaidopoulou Nteak, Gestur Vidarsson, Albert Bondt, Albert J. R. Heck

**Affiliations:** ^a^ Biomolecular Mass Spectrometry and Proteomics, Bijvoet Center for Biomolecular Research and Utrecht Institute for Pharmaceutical Sciences, Utrecht University, Utrecht 3584 CH, The Netherlands; ^b^ Netherlands Proteomics Center, Utrecht 3584 CH, The Netherlands; ^c^Sanquin Research and Landsteiner Laboratory, Department of Experimental Immunohematology, Amsterdam University Medical Center, Amsterdam 1066 CX, The Netherlands

**Keywords:** immunoglobulin A, J-chain, mass spectrometry, clonal repertoire, humoral immune response

## Abstract

Immunoglobulin A (IgA) is the most abundant antibody in humans, with high concentrations in both mucosae/secretions and circulation. While mucosal IgA has been studied extensively, characterization of human IgA in serum and its distinctive functions lags behind. Circulatory IgA is regularly assumed to be monomeric, despite some reports describing a minor population of J-chain-coupled dimers. Here, we first charted the compositional landscape of human serum IgA in individual healthy donors. In addition to the expected predominating monomers, we consistently observed J-coupled dimers, even representing ~30% of one donor’s total serum IgA. To determine whether these structurally distinct populations were also clonally distinct, we employed mass spectrometry–based IgA1 clonal profiling in sera of two donors. Our data revealed the majority of IgA1 clones are present solely as monomers, with a smaller number exclusively dimeric. Strikingly, a third population of IgA1 clones is present in circulation as both monomers and J-coupled dimers. In fact, these shared, structurally promiscuous IgA1 clones dominated both individuals’ serum IgA1 clonal repertoires. Our findings suggest every unique IgA1 clone could potentially be produced as a variable mixture of monomers and J-coupled dimers, but what exactly determines this ratio requires further investigation. This finding is important, as monomers and J-coupled dimers have distinct characteristics in antigen binding, receptor activation, and clearance from circulation, with several reports highlighting, for instance, the enhanced neutralization capacity of dimeric IgA. As we show here, the human immune system is not merely capable of producing both forms but apparently prefers doing so in parallel.

## Significance

Human immunoglobulin IgA occurs in diverse assemblies, mainly monomers (mIgA) and J-chain coupled dimers (dIgA). The general view is that these forms are produced at different sites, i.e., circulatory and mucosal, with mIgA assumed to represent the former. Inherently, no overlap would be expected between these populations’ clonal repertoires. Here, we report the protein-centric analysis of assembly-specific IgA1 clonal repertoires in sera of two healthy individual donors. Strikingly, we find a substantial subset of clones that co-occur as both mIgA1 and dIgA1 assemblies, hinting at common B cell origins and antigen reactivity. While further investigation is needed, these intriguing results nonetheless have potentially significant implications for production and understanding of human IgA.

Of all classes of immunoglobulins in humans, immunoglobulin A (IgA) is the predominant isotype found in the mucosal linings of the gastrointestinal, respiratory, and urogenital tracts and in external secretions such as tears, saliva, and colostrum/milk (1, 2). IgA is also prevalent in the lymphatic and circulatory systems, ranking as the second-most abundant Ig in adult serum (~0.5 to 3.5 mg/mL) after IgG (1). Together these high concentrations in both mucosal and systemic immune compartments make IgA the overall most abundant Ig in the human body (3).

**Fig. 1.**
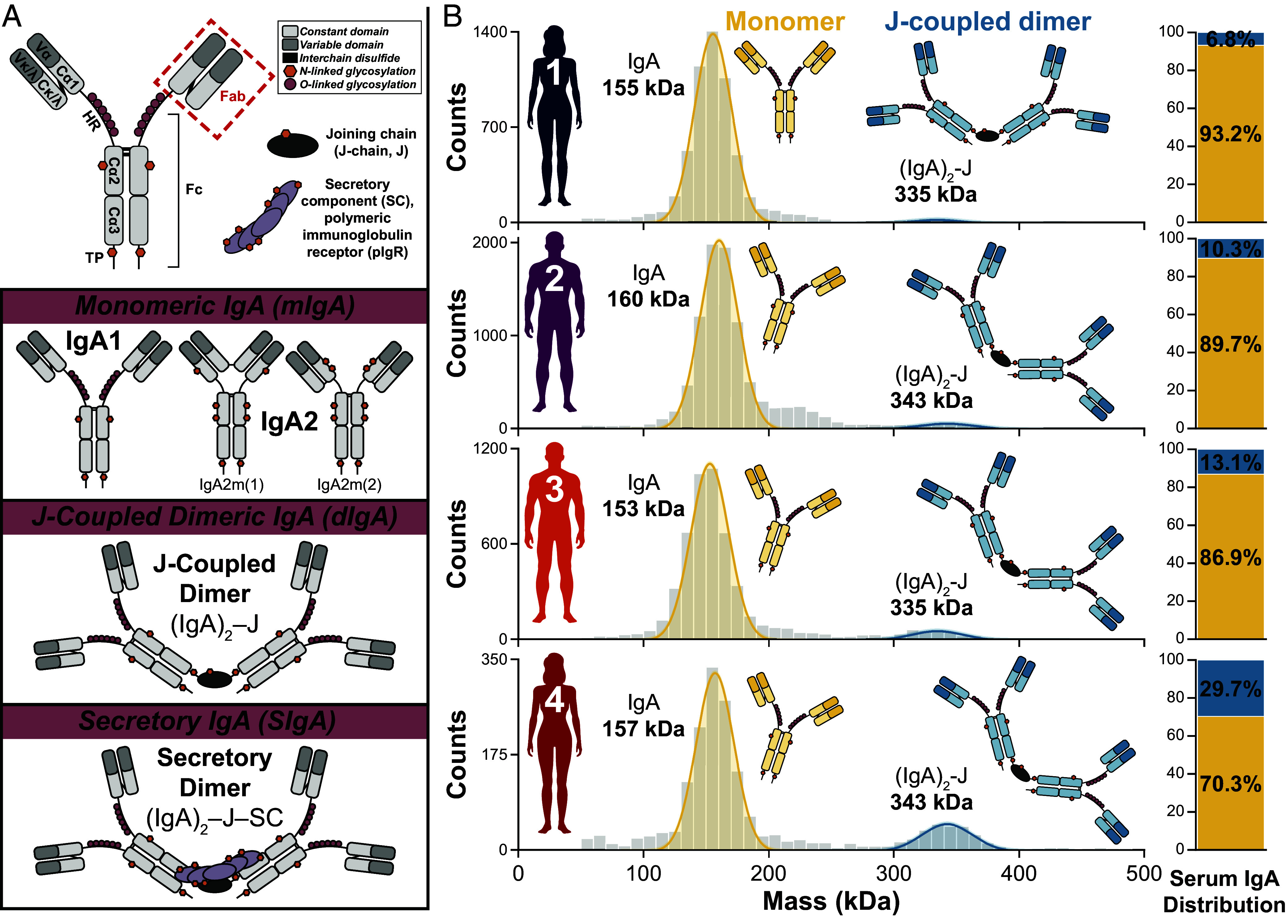
Human serum can contain a substantial amount of J-coupled dimers of IgA. (*A*) Schematic diagrams of human IgA1 monomer (*Top*) with domains, disulfide bonds, and glycosylation sites highlighted, along with the J-chain (J) and secretory component (SC) of pIgR assembly factors. Below are schematic models illustrating the molecular heterogeneity and structural variability of human IgA. The three distinct molecular forms are defined by the absence of both assembly factors (monomeric IgA, mIgA), presence of only J-chain without SC of pIgR (dimeric IgA, dIgA), and presence of both the J-chain and SC of pIgR (secretory IgA, SIgA) as indicated. Schematic models of monomeric human IgA1, IgA2m(1), and IgA2m(2) highlight the differences in structural features between the two subclasses and allotypes. (*B*) Mass distributions of the full repertoire of intact IgA assemblies purified from serum of four adult donors (Donors 1 to 4 as indicated) determined by mass photometry (MP). Two assemblies were detected in all four samples and assigned as monomers (yellow, ~160 kDa) and J-coupled dimers (blue, ~340 kDa). These assignments were verified by mass spectrometry-based bottom–up proteomics. The relative abundances of these assemblies varied between individuals, as indicated by the bar charts to the *Right* of each panel which depict the percentage of total circulatory IgA present as J-coupled dimers (blue) or monomers (yellow). The total number of individual monomer and J-coupled dimer molecules detected (represented by the color-shaded area of each assigned, fitted peak) were quantified and then adjusted accordingly to reflect the presence of two IgA monomer subunits in each J-coupled dimer.

All human Ig classes—IgM, IgD, IgE, IgG, IgA—have relatively similar domain structure due to their formation via class-switch recombination. They share the same basic protomer (H_2_L_2_) structure of two heavy (H) chains paired with two light (L) chains expressed and assembled by antibody-secreting cells (ASCs). IgG is present exclusively in this monomeric form in both serum and mucosal-associated biofluids/tissues. By contrast, human IgA is largely present as polymers in mucosal contexts. The presence of a C-terminal 18-residue peptide extension called the tailpiece (TP) in both IgA and IgM enables formation of J-coupled polymeric IgA or IgM (pIgA, pIgM) assemblies (4). This process occurs inside the ASC and depends upon incorporation of a single molecule of the immunoglobulin joining chain, or J-chain (J), prior to secretion of the fully assembled J-coupled polymeric complex, as free J-chain is not released (5, 6). While IgM exclusively assembles into J-containing pentamers which associate extracellularly with CD5L in the sera of healthy individuals (7), this degree of structural specificity induced by the J-chain is not mirrored in IgA. In vitro studies have demonstrated as many as five IgA protomers can be assembled alongside a single J-chain (8). However, such large structures are typically only found in trace amounts in humans—if detected at all (1). Instead, pIgA is by far predominantly found in the form of J-coupled dimers (dIgA) (2), which will be the central focus of the present work (Fig. 1*A*). Regardless of the stoichiometry, the presence of the J-chain in polymeric assemblies of IgA or IgM enables binding to the polymeric immunoglobulin receptor (pIgR) essential for transepithelial transport into the mucosae (9). There, proteolytic cleavage of pIgR releases the J-coupled dIgA into the mucosal lumen with part of pIgR’s N-terminal extracellular domain—called the secretory component (SC)—still attached. Together, the pIgA-J-SC complex constitutes a distinct molecular form (secretory IgA, SIgA) which is specific to mucosal contexts (Fig. 1*A*). There, it forms the first line of defense in protecting the intestinal epithelial barrier from enteric infection and invasion by pathogenic microorganisms (1).

Incorporation of the J-chain thus gives rise to aspects of human IgA’s uniquely extensive molecular heterogeneity (10). Moreover, J-chain association is merely optional rather than required for production of human IgA, which consequently exhibits structural promiscuity whereby both monomeric IgA lacking the J-chain (mIgA) and J-coupled polymeric forms (including dIgA) naturally occur—a uniquely distinct feature compared to all other human Ig isotypes. With two subclasses (namely IgA1 and IgA2, Fig. 1*A*) found only in humans and some other great ape species, the human IgA system is also distinct compared to most other mammalian IgA (11). Human IgA1 and IgA2 are highly similar with ≥90% sequence homology between the three Cα domains (2). Differences are almost exclusively restricted to the hinge region (HR) which is 13 amino acids longer in IgA1, featuring two Pro/Ser/Thr-rich repeats that introduce numerous putative O-glycosylation sites exclusively to this subclass (Fig. 1*A*). While the extended IgA1 hinge confers greater conformational flexibility, it also makes IgA1 susceptible to cleavage by specific bacterial proteases. Further structural and functional differences between IgA subclasses arise due to their distinct glycosylation profiles (12–15). The distribution of subclasses as well as molecular composition varies throughout the body (16).

Given the dominance of IgA at mucosal surfaces and in secretions, human IgA has been extensively characterized in the context of mucosal immunology (i.e., SIgA) (8, 9, 17). The functions of human IgA in serum are less well characterized. While the structural and molecular composition is clearly distinct from that of mucosal IgA, several basic details still have not been exactly defined in serum (18, 19). Such characterization is especially lacking in the context of healthy individuals, with IgA nephropathy and other pathologies the focus of most research efforts (20–29). Serum IgA is dominated by the IgA1 subclass and is often assumed (or implied) to be exclusively monomeric and thus devoid of the J-chain (2, 9, 19, 26, 27, 30–34). However, a few studies have reported the presence of a second, minor population corresponding to J-coupled dimers, but with discrepancies in the reported fractional abundances which range from negligible (<1%) up to ~22% of total serum IgA (10, 29, 35). Whether such values refer to bulk population measurement or were derived from observations within a single donor is often unclear. With nearly no pIgR present in serum, these J-coupled dimers of IgA in circulation are devoid of this cofactor and thus distinct from SIgA.

Serum IgA, like any Ig, in fact represents a molecular mixture of thousands to millions of different IgA1 clonal variants (36). Each of these has a unique amino acid sequence in the variable regions contained within the Fab arms that dictates antigen specificity and reflects their distinct cellular origins (37). We previously found that endogenous IgG1 and IgA1 repertoires are often dominated by just hundreds of unique clones (38–40). Whether these dominant IgA1 clones correspond to mIgA1 or dIgA1 is completely unknown but critical to fully understand human IgA. Not only do monomers and J-coupled dimers exhibit structural and functional differences, but they have also been suggested to originate from distinct J− and J+ IgA+ ASC populations located in distinct immune compartments (34, 41).

Here we aim to decipher the exact molecular and clonal composition of human serum IgA1 in individuals under healthy conditions. Using single-molecule MP and mass spectrometry (MS)–based proteomics to identify IgA assemblies purified from sera of four healthy individual donors, we chart the varied compositional landscape of human serum IgA. For two of these healthy donors, we further utilize a MS-based approach to monitor endogenous IgA1 Fab molecules from each assembly population (mIgA versus dIgA) separately. We report the assembly composition of human serum IgA1 with clonal resolution, annotating the intact molecular form(s) for nearly 3,000 distinct endogenous IgA1 clones. Importantly, we reveal clone-specific features of IgA’s inherent structural promiscuity related to J-chain incorporation. These findings potentially have significant implications for understanding the cellular origins, functions, and complexity of human circulatory IgA.

## Results

### In Healthy Individuals, Up to One-Third of Serum IgA Can Be Present as J-Coupled Dimers.

The first unresolved question we aimed to address was whether human circulatory IgA exists exclusively as monomers or exhibits widespread structural promiscuity. All possible molecular forms of circulatory IgA—i.e., mIgA and pIgA assemblies lacking SC—can readily be discriminated from each other by mass and composition defined by the absence or presence of J-chain, respectively (Fig. 1*A*). We therefore investigated the molecular composition of intact IgA assemblies affinity-purified from the serum of four healthy adult donors (Donors 1 to 4, *SI Appendix*, Table S1) with single-molecule MP. The resulting MP mass histograms (Fig. 1*B*) depict the distribution of all detected particles, allowing identification of each species by mass and quantitative comparison between co-occurring populations.

The distribution of circulatory IgA assemblies from each of the four healthy donors featured as expected a dominant peak at ~160 kDa, assigned as IgA monomers (Fig. 1*B*). We also detected the presence of J-coupled dimers at ~340 kDa for all four donors. We highlight the absence of larger assemblies in circulation, as evidenced by the lack of any peaks consistent with the associated theoretical masses (40). These assignments are further supported by bottom–up MS-based proteomics analysis of these samples (*SI Appendix*, Fig. S1*A*), confirming the presence of J-chain and lack of pIgR SC.

As expected, J-coupled dimers were less abundant than monomers in all four donors (Fig. 1*B*). However, the intact assembly distribution varied considerably, with roughly 7%, 10%, 13%, and 30% of the total IgA of Donors 1 to 4, respectively, present as J-coupled dimers without SC of pIgR. For this quantitative analysis, we adjusted the particle counts considering that each dIgA molecule detected contains two copies of IgA monomer subunits (*SI Appendix*, Table S2 and *Supplementary Materials and Methods*). The assembly distributions of human IgA in serum reported in the literature vary substantially, with J-coupled dimers accounting for anywhere from as low as <1% up to as high as ~22% of all circulatory IgA (10, 29, 35). Although some reports note the possibly high occurrence of serum dIgA in various pathologies (28, 29), the more common view seems to be that there is no (or effectively zero) dIgA in circulation in healthy conditions, based on recurrent descriptions of serum IgA as predominantly—or even exclusively—monomeric often with no mention of J-coupled dimers (2, 9, 19, 26, 27, 31–34). Although we analyzed only four donor samples here, we observed a substantial amount of dIgA in each, indicating that J-coupled dimers are more prevalent in the circulation of healthy individuals than often assumed.

To further contextualize results from these four donors, we analyzed a publicly available plasma proteomics dataset (42) of 687 samples from 139 individuals for the abundances of IgA, IgM, J-chain, and CD5L. Assuming that all J-chain protein detected in circulation—in accordance with the known requirements for proper folding and secretion (5, 6, 43)—must be in complex either with IgM pentamers associated with CD5L (IgM_5_-J-CD5L) or with dIgA (IgA_2_-J) (7, 44), the estimated relative abundance of dIgA was derived from the MaxLFQ values of these four proteins, as commonly used in quantitative proteomics (7, 45–47). Using this approach described in greater detail in *SI Appendix*, *Supplementary Materials and Methods*, we observed that the fractional abundance of J-coupled dimers also varies extensively between the hundreds of individual donors in this dataset (42), which indeed ranges from as low as <1% up to as high as ~33% of the total serum IgA (*SI Appendix*, Fig. S1*C*), validating our data. We also highlight the close agreement between the IgA assembly distribution determined with MP and the values calculated from bottom–up proteomics analysis of the four donors’ serum/plasma (*SI Appendix*, Fig. S1*C*).

### Monomers and J-Coupled Dimers of IgA1 Are Clonally Diverse in Human Serum.

**Fig. 2.**
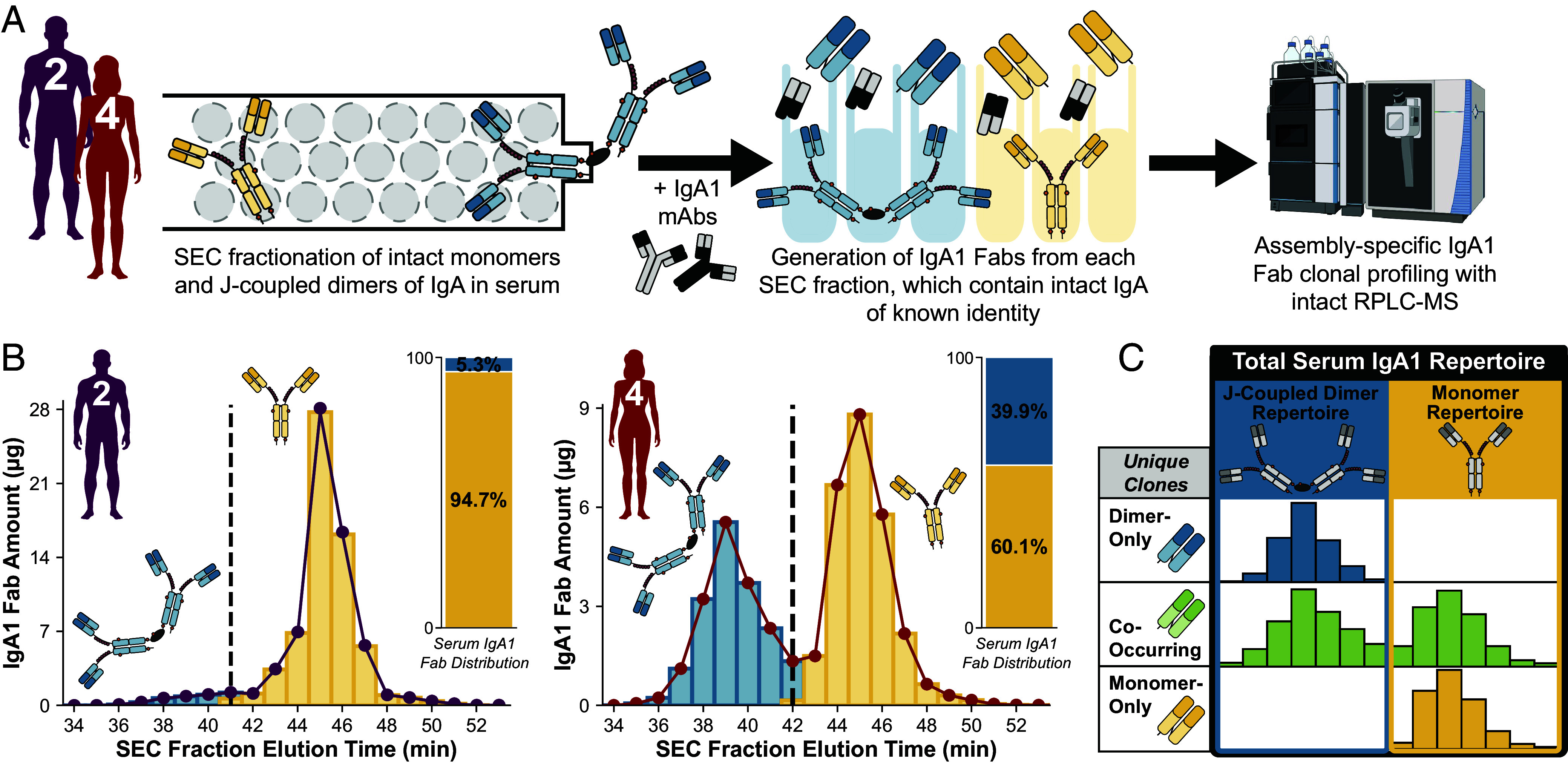
Assembly-specific profiling of IgA1 clonal repertoires reveals structural promiscuity of individual clones. (*A*) Approach taken for the profiling of IgA1 Fab clonal repertoires. Size exclusion chromatography was used to separate intact monomer and J-coupled dimers present in serum, followed by generation of IgA1 Fabs from each monomer- or dimer-containing SEC fraction individually. After spiking in two recombinant monomeric IgA1 mAbs into each SEC fraction to enable intensity-based quantitation, all IgA was captured with an affinity resin, which was then washed to remove unbound non-IgA proteins. Digestion with an O-glycan-specific protease resulted in the release of IgA1 Fabs from each SEC fraction, which were subsequently analyzed by intact RPLC-MS. (*B*) Cumulative IgA1 Fab abundance detected and quantified by intact RPLC-MS in each SEC fraction sample, effectively reconstructing the SEC elution profiles of the intact circulatory IgA1 assemblies for Donor 2 (*Left*) and Donor 4 (*Right*). Peaks are annotated as monomers (yellow) or J-coupled dimers (blue), and the inset bar chart depicts the cumulative abundance of all detected IgA1 Fabs from each of the two assemblies. (*C*) Visual reference for key concepts and terminology used. Most unique Fab clones originated exclusively from monomers (“monomer-only,” yellow), with a smaller but appreciable number exclusively found as J-coupled dimers (“dimer-only,” blue). An intriguing third subset of unique IgA1 clones co-occurred in circulation in both intact monomer and J-coupled dimer forms simultaneously (“co-occurring,” green). These three clone populations comprise the “total” serum IgA1 repertoire (black box) which can also be divided into two assembly-specific “J-coupled dimer” (blue box) and “monomer” (yellow box) repertoires as indicated in the illustration.

Our next aim was to dissect endogenous circulatory IgA1 at the protein level with clonal resolution to determine whether the monomeric and J-coupled dimeric repertoires have shared clonality or are mutually exclusive. We previously established methods for profiling endogenous IgA1 Fabs in serum as well as in breastmilk but did not probe their precise molecular composition in these studies (38–40). Here, we adapted this earlier approach for the assembly-specific characterization of endogenous circulatory IgA1 clonal repertoires (Fig. 2*A* and *SI Appendix*, Fig. S2). Details regarding the optimization and experimental validation of our MS-based approach to IgA1 Fab clonal profiling can be found in previous publications (38–40), as also summarized in *SI Appendix*, *Supplementary Materials and Methods*.

The circulatory IgA of Donors 1-4 were all similarly dominated by the IgA1 subclass (≥85%) based on MS-based proteomics and therefore suitable for IgA1-based repertoire analysis (*SI Appendix*, Fig. S1*B*). From the four individuals, we selected Donors 2 and 4, who had a normal (~10%) and high (~30%) proportion of dIgA, respectively (Fig. 1*B*), for more detailed clonal analysis. We used offline size exclusion chromatography (SEC) to separate intact monomeric and J-coupled dimeric IgA assemblies in the serum of Donors 2 and 4 (Fig. 2*A*). Each donor’s intact J-coupled dimers and monomers of IgA were collected in discrete sets of fractions (Fig. 2*A*). Single-molecule MP analysis of SEC-fractionated affinity-purified IgA provided mass-based evidence for the elution range of each intact assembly (*SI Appendix*, Fig. S2*A*), with the apexes of the peaks associated with dIgA and mIgA separated by five fractions. MS-based proteomics analyses of all fractions from SEC separation of both affinity-purified IgA and crude plasma confirmed the range over which IgA and J-chain coelute as J-coupled dimers (*SI Appendix*, Fig. S2 *B* and *C*). J-chain peptides are notably absent from monomeric and later-eluting fractions, thereby confirming the absence of free J-chain in serum.

With the intact form of IgA present in each SEC fraction thus clearly defined based on elution time, we generated IgA1 Fab molecules for each IgA-containing SEC fraction individually (Fig. 2*A*) as detailed further in *SI Appendix*, *Supplementary Materials and Methods* (38, 40). Using this approach, distinct IgA1 Fab clones are identified by their characteristic unique mass and reversed phase liquid chromatography (RPLC) retention time (RT), and the abundance of each of these unique clones in each fraction is determined. This allows reconstruction of the intact IgA1 SEC elution profile—using the cumulative detected abundance as in Fig. 2*B* or that of a single distinct clone—and assignment of assembly state, i.e., mIgA1 or dIgA1 (Fig. 2*B*). As expected, the cumulative fractional abundances of Fabs originating from intact IgA1 monomers or separately from J-coupled dimers (Fig. 2*B*) differed considerably between Donors 2 and 4, in line with their intact assembly distributions observed by MP (Fig. 1*B*). Also as anticipated, the unique IgA1 clones present as monomers outnumbered the unique J-coupled dimer clones, and this difference was markedly higher for Donor 2 than for Donor 4 (*SI Appendix*, Fig. S3). Importantly, we found both the monomer and J-coupled dimer populations to be unique and clonally diverse, with hundreds of distinct IgA1 clones identified for each assembly state in both donors (*SI Appendix*, Fig. S3 *A* and *B*). Further, with respect to Fab mass and RPLC RT, we observed similar distributions between both assembly-specific clonal populations—i.e., the variable Fab regions from mIgA1 versus dIgA1 antibodies do not fall within obviously distinct mass windows or RT ranges (*SI Appendix*, Fig. S3*C*).

### Structurally Promiscuous Clones Dominate Healthy Human Serum IgA1.

By using SEC fractionation prior to IgA1 clonal profiling, we were able to extend the depth of our analysis considerably, whereby the resulting total serum IgA1 repertoires contained more than 1,400 unique clones per donor. For each of the nearly 3,000 unique endogenous IgA1 Fab clones identified in total from the two donors analyzed, we obtained individual SEC elution profiles and thus were able to determine each clone’s assembly distribution. All results from this analysis are summarized in *SI Appendix*, Table S3.

The majority of unique clones were present solely as IgA1 monomers, with considerably fewer (but still >100 for each donor) found exclusively as J-coupled dimers (*SI Appendix*, Table S3). In addition to these “extremes,” in both donors we also observed hundreds of unique clones that co-occurred in both mIgA and dIgA forms, readily evidenced by their appearance (green markers) at the same RPLC RT-Fab mass coordinates in the dIgA1 and mIgA1 repertoires shown in *SI Appendix*, Fig. S3 *A* and *B*. Thus, human serum does not simply contain two distinct IgA1 clonal repertoires corresponding to separate pools of monomers and J-coupled dimers. Instead, the “total” serum IgA1 repertoire is divided into three subpopulations of unique clones, hereafter referred to as “monomer-only”, “dimer-only”, and “co-occurring” and color-coded throughout this manuscript by yellow, blue, and green, respectively (Fig. 2*C*). We also define two assembly-specific “monomer” and “dimer” clonal repertoires (*SI Appendix*, Fig. S3 *A* and *B*, respectively). The relationship between these repertoires (total, monomer, dimer) and unique clone (monomer-only, dimer-only, co-occurring) classifications is illustrated schematically in Fig. 2*C*.

Consistent with previous analyses of IgG1 and IgA1 Fab clonal profiles (38–40), both donors’ total and assembly-specific serum IgA1 repertoires are relatively simple, dominated by tens to hundreds of highly abundant clones. In more detail, just ~5% of clones collectively contribute ~50% of the total IgA1 abundance in each repertoire (*SI Appendix*, Fig. S4). Here, we highlight this trend holds true even though we detect significantly more distinct clones (~3.5-fold, >1,400 per sample) with improved sensitivity owing to the additional SEC-based separation, as compared to previous IgA1 Fab clonal profiling in serum (40).

**Fig. 3.**
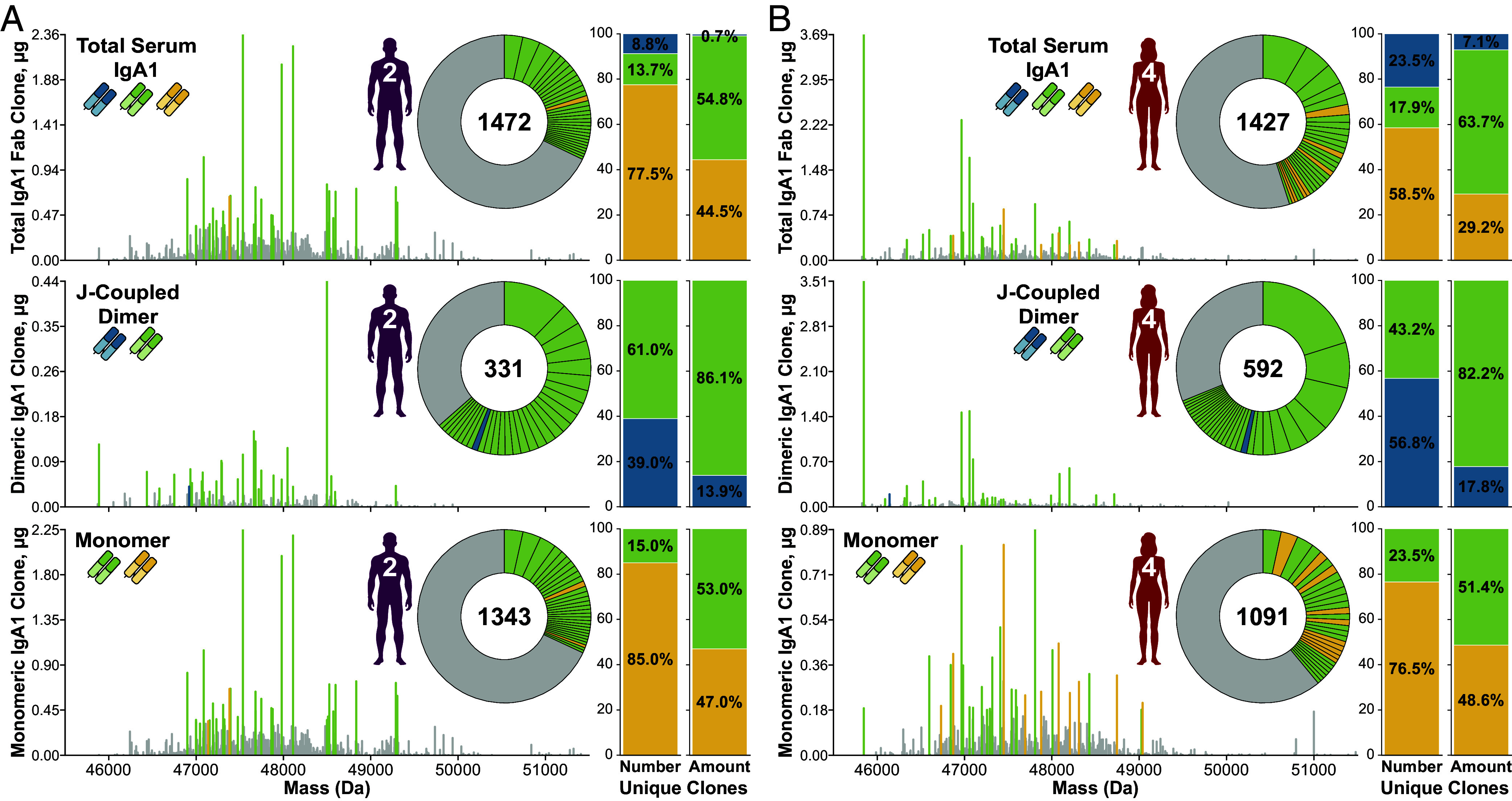
A small proportion of unique clones present in both monomer and J-coupled dimer forms dominates serum IgA1 repertoires. Total (*Top*), J-coupled dimer (*Middle*), and monomer (*Bottom*) circulatory IgA1 clonal profiles for Donor 2 (*A*) and Donor 4 (*B*). Each stick represents a unique IgA1 Fab clone, plotted according to its characteristic mass and abundance. The top 30 most abundant clones in each panel are annotated as monomer-only (yellow), dimer-only (blue), or co-occurring (green), with their fractional abundance indicated by the relative size of the colored wedge slices of the inset donut chart. All clones ranking outside the top 30 most abundant are represented by gray. The total number of unique IgA1 Fabs detected in each repertoire is indicated at the center of the inset. Bar charts to the *Right* of each plot indicate the proportion of the total number of unique clones (*Left* bar chart in each pair) and fractional abundance (*Right* bar chart in each pair) of unique clones in the indicated circulatory IgA1 repertoire contexts.

Remarkably, of the three types of unique clones revealed here, the most dominant clones are largely part of the co-occurring clonal population (Fig. 3). In the total serum IgA1 clonal repertoires of Donors 2 and 4 (Fig. 3 *A* and *B*, respectively), by their numbers less than 20% of all unique clones are classified as co-occurring, and yet as a group their cumulative fractional abundance accounts for over 50% of the total circulatory IgA1.

Likewise, J-coupled dimer repertoires are dominated by clones co-occurring in the monomeric fraction rather than by those found exclusively as dIgA (Fig. 3). Although they contain fewer unique clones (*SI Appendix*, Fig. S3*C*) and lower concentrations than the monomer and total serum IgA1 repertoires (*SI Appendix*, Table S3), these J-coupled dimeric IgA1 repertoires are especially “simple.” Indeed, a staggering ~60 to 70% of the total abundance is contributed by just the top 30 clones, i.e., fewer than one-tenth of all unique clones occurring in J-coupled dimer form (Fig. 3 and *SI Appendix*, Fig. S4). This is particularly pronounced for Donor 4, for whom just the top seven most dominant clones account for half of the total J-coupled dimer amount (Fig. 3*B*). Despite Donor 4’s dimer-only clones (336) alone outnumbering Donor 2’s entire dimer repertoire (331), the composition of both donors’ top 30 dIgA clones is highly similar–i.e., all but one of these top dimer clones were also found as monomers without the J-chain. Thus Donor 4’s “extra” dimer-only clones have very low abundances (Fig. 3). Differences between the donors’ intact IgA assembly distributions observed by MP (Fig. 1*B*) therefore are not explained by the significant disparity in the number of unique clones.

### Assembly Composition Varies between Individual IgA1 Clones.

**Fig. 4.**
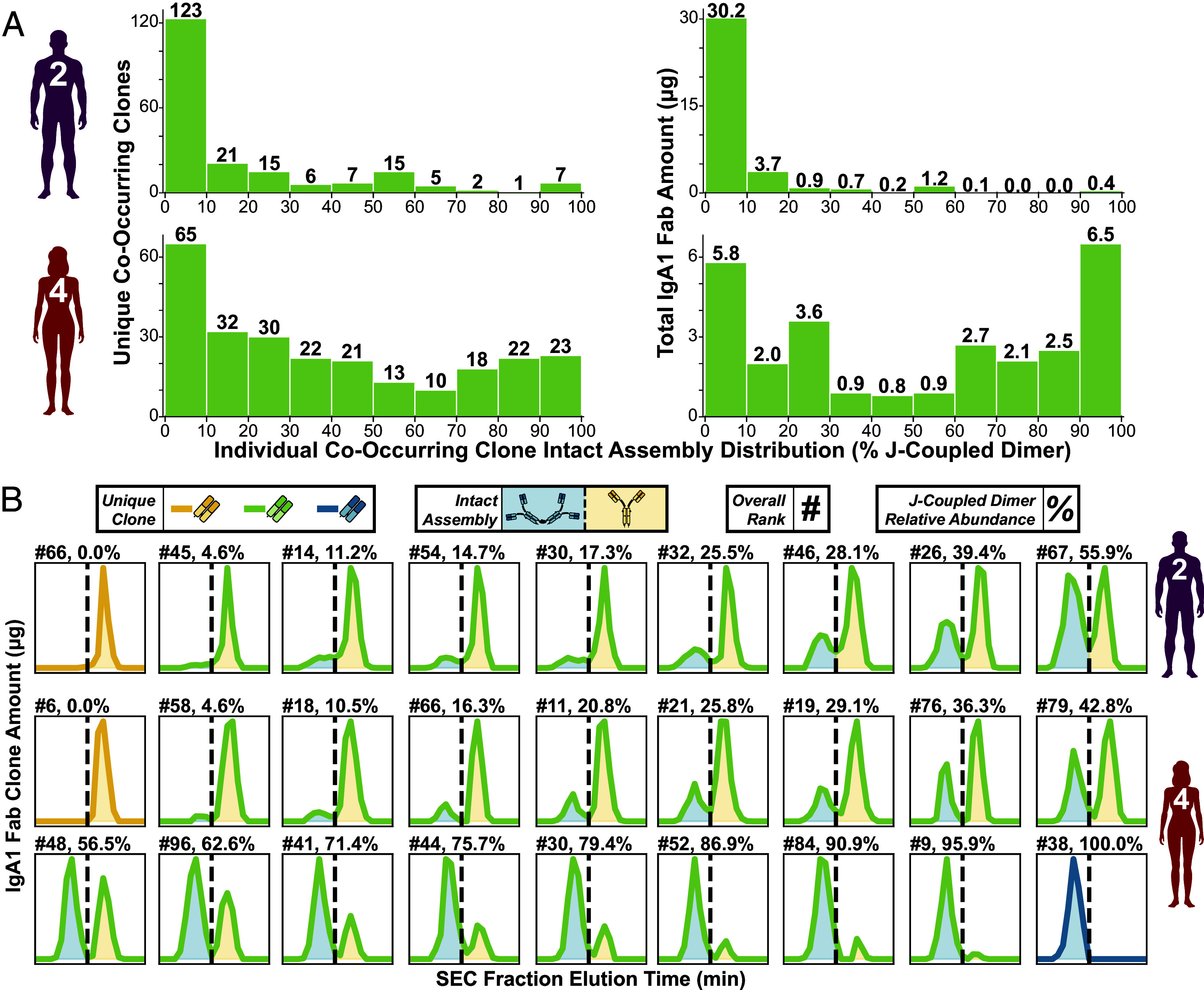
The assembly distribution of distinct co-occurring IgA1 clones varies widely. (*A*) Histograms depicting the assembly distribution of all unique co-occurring IgA1 clones in Donor 2 (*Top*) and Donor 4 (*Bottom*) according to number (*Left*) and cumulative abundance (*Right*). The ten bins in each histogram are defined according to the relative intact assembly distribution (i.e., the fractional abundance of J-coupled dimer assemblies) of those clones. (*B*) SEC elution profiles of distinct co-occurring IgA1 Fab clones selected together with one monomer-only and one dimer-only clone (if applicable) from the top 100 overall most abundant clones in the total serum IgA1 repertoire of Donor 2 (*Top* row) and Donor 4 (*Bottom* two rows). Each panel is annotated with the clone’s overall donor-specific ranking (#) and fractional J-coupled dimer abundance (%). Plots are shown in order of increasing J-coupled dimer fractional abundance (%). Line color indicates the corresponding unique clone type according to the color scheme shown in the legend, with the area under the peaks shaded according to the discrete elution ranges of intact J-coupled dimers (blue) and monomers (yellow). Bimodal distributions directly indicate the clone’s co-occurrence in both forms.

Each donor’s cumulative IgA1 Fab abundance is differently distributed between the two assemblies (Fig. 2*B*) and among the three unique clone classes (Fig. 3). This indicates that the structurally promiscuous IgA1s co-occur in different mIgA/dIgA assembly ratios. In particular, the J-coupled dimeric form must correspond to a much larger fraction of the total co-occurring clone abundance for Donor 4 than for Donor 2. Indeed, monomers account for >90% of the cumulative co-occurring clone abundance for Donor 2 (*SI Appendix*, Fig. S5*A*). In contrast, in Donor 4 they are nearly equal with their J-coupled dimer counterparts overall (*SI Appendix*, Fig. S5*A*). However, each unique co-occurring clone could have a varying distribution that does not match these overall trends, with some being predominantly monomeric, but other clones more dimeric (*SI Appendix*, Fig. S5). We therefore next compared how the assembly ratios of each donor’s individual clones were distributed. For Donor 2, this distribution is heavily skewed toward the monomer-dominated end, compared to the relatively more even distribution for Donor 4 (*SI Appendix*, Fig. S5*B* and Fig. 4*A*). Clearly, co-occurring clone assembly composition is not a donor-specific feature but instead appears to be clone-specific. This variation is best illustrated by plotting the SEC profiles of individual distinct IgA1 clones identified by their unique Fab mass/RPLC RT signature and quantified in each fraction (Fig. 4*B*). The appearance of a bimodal distribution is diagnostic for co-occurring clones in accordance with the established discrete elutions of intact J-coupled dimers and monomers. Examples selected from each donor’s top 100 most abundant IgA1 clones are shown in order of increasing J-coupled dimer fractional abundance (Fig. 4*B*). This reaches a maximum of ~56% J-coupled dimer for Donor 2, while the top 100 most abundant IgA1 co-occurring clones from Donor 4 already illustrate the full extent of variation possible (Fig. 4*B*).

We conclude from the data that each unique IgA1 clone in circulation could be present in a mixture of assembly states varying from solely monomeric to solely J-coupled dimeric (*SI Appendix*, Fig. S5). In between these extremes and dominating the total serum repertoire are IgA1 clones that in circulation simultaneously occur both with and without J-chain, revealing that the structural promiscuity of human IgA is not just a bulk population property—at least in these two donors. The observation that each discrete IgA1 clone—defined by its unique IgA+ ASC origin—has a unique assembly distribution is, as far as we know, unprecedented and raises several compelling research questions we address in the Discussion.

## Discussion

In the adaptive humoral immune response, binding of an antigen to the B cell receptor induces naïve B cells to proliferate, undergo affinity maturation, and ultimately differentiate into an ASC capable of producing massive quantities of antigen-specific antibody. Each Ig sequence derived from a different B cell is not only unique but also directly reflects its clonal origins and unique antigen target (48, 49). A view commonly presented in IgA literature is that J-coupled polymeric IgA and monomeric (i.e., non-J-coupled) IgA—which dominate mucosal and systemic sites, respectively—originate from two distinct populations of IgA+ ASCs localized to the gut and bone marrow, respectively, in which J-chain is expressed (J+) or absent (J−) (43). Accordingly, monomers and J-coupled dimers of IgA should correspond to two distinct clonal repertoires with no overlap in cellular origins or antigen binding.

This clearly does not align with the current findings. We observed hundreds of unique co-occurring clones in the two donors’ serum IgA1 repertoires despite significant differences in their overall intact assembly distributions (Fig. 1*B*). Moreover, in both cases these structurally promiscuous clones exhibited the same major features, making up the majority of the total circulatory IgA1 pool (Fig. 3) and spanning the full range of possible co-occurring assembly ratios (Fig. 4). These results have potentially significant implications for our understanding of human IgA but are currently limited to the small cohort of this study. Analysis of a larger, more diverse donor cohort is needed to better define the boundaries of the compositional landscape of healthy human serum IgA and to determine the prevalence of co-occurring clones.

### Evaluation of Potential Methodological Biases.

In our protein-centric approach, each unique IgA1 clone is defined by its unique combination of Fab mass and RPLC RT signature, independent of the assembly origin(s) defined by SEC fraction RTs as discussed below. We previously validated the use of this combined identifier as a proxy for Fab sequence (39, 40). Any differences in sequence—and thus differences in clonality and antigen reactivity—are inherently reflected in the Fab mass, with even coincidentally isobaric masses discriminated by RPLC RT. Both the mass and apex RPLC RT must match within tolerances of ±2 Da and ±1 min to assign shared clonality, which we note is more stringent than “clonal relatedness” defined by matching V and J gene usage with similar (but not strictly identical) CDR3 sequences (34, 50). In clonal mixtures of below a few thousand, as analyzed here, the probability of two unrelated Fabs meeting these criteria by random chance is negligible, as supported by our consistent observation that Fab clonal repertoires are highly unique with virtually no overlap between individuals (25, 38–40). On the other hand, monomeric and J-coupled dimeric forms of the same unique IgA1 sequence could conceivably be differentially posttranslationally modified, but nearly all PTMs induce significant mass shifts that already fall well outside the accepted window regardless of associated RPLC RT differences. While correct assignment of assembly origins would not be impacted, such discrepancies in PTM of mIgA and dIgA would ultimately result in misclassification of the corresponding Fabs as distinct monomer-only and dimer-only clones and therefore also in underestimation of the co-occurring clone population.

All Fab molecules detected in a particular SEC fraction are considered to originate either from dIgA or from mIgA assemblies according to the multifraction elution ranges of intact monomers and J-coupled dimers of IgA (Fig. 2*B* and *SI Appendix*, Fig. S2). (To be clear, SEC fraction RTs—used to assign intact assembly origins—and RPLC RTs—used in conjunction with Fab mass to assign shared clonality—are discrete.) Coelution of dIgA and mIgA within the same SEC fraction due to, e.g., peak broadening/tailing causing overlap, could therefore result in artificial co-occurring clone assignment. Individual unique IgA1s could possibly deviate from the fractionation scheme defined for the bulk intact assembly populations due to, e.g., glycosylation heterogeneity and/or structural conformations. However, the SEC profiles of individual unique clones shown in Fig. 4 provide clear, strong evidence of the structural promiscuity of distinct IgA1s in circulation, which varies widely between clones. Bimodal distributions are diagnostic for co-occurring clones, directly reflecting the serum dIgA1 and mIgA1 assembly origins of the detected Fab molecules. SEC separation fidelity is clearly demonstrated at clonal resolution by these profiles, validating our approach and substantiating the phenomenon of co-occurring clonality.

However, the mIgA1/dIgA1 assembly ratios determined for individual unique IgA1 clones may differ from their true values. Assembly distributions are calculated assuming the hinge regions of monomers and J-coupled dimers are cleaved with equal efficiency, validated by previous experiments (38). Unequal Fab release from monomers and J-coupled dimers could still occur for some individual unique IgA1 clones, inflating the apparent abundance of one form over the other and resulting in skewed assembly distributions. Differences in hinge O-glycosylation—especially between assembly states of the same unique IgA1 clone—could also result in skewed assembly ratios due to differences in cleavage site or multiple cleavage variants. However, we have previously shown less than one-third of all unique endogenous IgA1 clones have a missed cleavage variant, which is detected in minor amounts compared to the main Fab (38).

Monomers have enhanced half-lives in serum while J-coupled dimers have higher serum clearance rates and can potentially also be transported out of serum by using pIgR (3, 9, 14, 30). Together this makes detection of monomeric IgA more likely. The hundreds of dimer-only clones and predominantly dimeric co-occurring clones observed (Fig. 4 and *SI Appendix*, Fig. S5) therefore strongly support the validity of our findings. It could also be expected that some monomer-only clones may have been co-occurring and the relative abundance of co-occurring clones in the form of mIgA1 may be falsely inflated. The presence of some unique IgA1 clones may not have been detected in all relevant SEC fractions due to differences in dynamic range and limits of detection. This could result in some co-occurring clones inaccurately being classified as monomer-only or dimer-only.

A limitation of our protein-centric approach is that the cellular origins of these co-occurring IgA1 antibody clones cannot be directly established. This introduces the possibility that the structural promiscuity and assembly ratios observed at the protein level may not be the product of IgA+ ASCs but instead arise due to extracellular dynamics. However, while postsecretory dissociation of J-coupled dimers and/or extracellular association of monomers is theoretically possible, this is not supported by the current data or other reports (5, 51–54). The J-chain, with 8 cysteines, does not form stable, defined disulfide structures on its own without IgA/IgM and is consequently not secreted but instead retained intracellularly before eventually being degraded (55, 56). Free J-chain is thus not normally expected in serum but would be released from extracellular dissociation of J-coupled dimers and is required for their assembly from monomers. Results from bottom–up proteomics analysis of SEC-fractionated donor plasma (*SI Appendix*, Fig. S2*C*) show there is no detectable free J-chain present in circulation. The SEC elution profile of J-chain in crude serum/plasma (*SI Appendix*, Fig. S2*C*) coincides strictly with IgM (i.e., in J-coupled IgM pentamers which extracellularly associate with CD5L) and with IgA’s earlier-eluting, higher MW peak which corresponds to J-coupled dIgA, as we have also shown previously (40). We therefore conclude the observed co-occurring assemblies are more likely to reflect ratios from production by IgA+ ASCs rather than extracellular dynamics.

### Alternative Explanations for Production of Co-Occurring Assemblies of IgA1 Clones.

The simplest explanation for the current data is that co-occurring mIgA1 and dIgA1 assemblies correspond to identical Fabs with distinct clonal origins. Distinct ASCs with identical convergent V(D)J rearrangements could exist, with some secreting monomers and others secreting J-coupled dimers. Convergent antibody signatures have been observed between individuals in response to various pathogens and vaccinations, but these “public” clonotypes account for a very small fraction of the total repertoire (for IgA, 0.5% or less) (50, 57). However, the likelihood of this is much higher within the same donor due to individuals having different gene use biases that are remarkably stable over time (57, 58). Indeed, identical V(D)J rearrangement in multiple individual B cells within the same donor was recently reported, corresponding to ~1% of all unique sequences identified in total across the six-donor study (50). These cells appeared to have undergone extensive clonal expansion without hypermutation of their still-identical sequences and were colocalized not just within one specific tissue but were distributed nearly universally throughout all organs tested (50). This phenomenon was most prevalent among proliferative “plasmablast-like” ASCs especially dominant in peripheral blood and among “older” B cell lineages with higher hypermutation frequencies, indicating significant involvement in persistent systemic responses to antigen (50). Human bone marrow plasma cells (PCs) have been proposed to originate in part from reactivated circulating memory B cells (MBCs) that could similarly proliferate and differentiate into clonally expanded ASCs without accruing new sequence mutations (59). Of particular note, J-chain expression was reportedly higher in such identical, lowly mutated MBC clones as compared to clonally related—but not identical—PCs resulting directly from B cells that underwent somatic hypermutation in germinal center reactions (59). Collectively, findings from these studies illustrate plausible scenarios whereby highly abundant identical IgA1 antibody clones in serum could arise from diverse cellular phenotypes and origins.

The possible explanation above requires the assembly output to differ between these distinct ASCs with identical V(D)J rearrangements, which could be attributed to some being J+ and others J−. However, as summarized nicely by Castro and Flajnik (43), the persistent view that circulatory IgA-producing ASCs in the bone marrow are J− (60) is largely based on early studies that very likely suffered from technical limitations and inadvertently underestimated the amount of J-chain. Based on more recent advances, the J-chain is now considered to be a marker of B cell differentiation into ASCs (61–65). Although it is known that assembly and secretion of J-coupled pIgA is posttranslationally controlled (66–70), there are still many open questions regarding how J-chain expression is regulated (43)—including whether expression might be spatiotemporally dynamic—and the exact details of the assembly mechanism of J-coupled polymeric IgA, which must differ from that of J-coupled IgM pentamers (55, 71–73).

Another possible explanation for the presence of monomers and J-coupled dimers of IgA1 with identical clonality in circulation is their coproduction by the same cell. Individual IgA+ ASCs that coexpress J-chain could be engaged in the simultaneous synthesis of both J-devoid monomeric and J-coupled dimeric IgA to differing degrees (74, 75), in line with mIgA/dIgA ratios varying widely between distinct co-occurring clones in serum as reported in this study as well as by Park et al. (76). This was originally suggested several decades ago from in vitro experiments and has been shown for individual hybridoma and myeloma cell lines (54, 74, 75, 77). Secretion of exclusively one form or different ratios of both mIgA and dIgA assemblies could depend on the level of J-chain in these individual cells, all else being equal. Many different groups have shown that the distribution of mIgA, dIgA, and larger pIgA assemblies produced recombinantly is directly influenced by the relative amounts of J-chain, IgA heavy chain, and light chain DNA/transcripts (30, 54). In vitro experiments have indicated that the intracellular pool of J-chain is the limiting factor (54), with a threshold minimum concentration—not simply just a nonzero amount of J-chain available—required for dIgA assembly (53). In line with this, results from recent single-cell RNA sequencing studies show variation in the levels of J-chain, both absolute and relative to that of the heavy chain (61).

We note that the J-chain expression is a necessary but not sufficient condition for assembly of J-coupled dimers and polymers of IgA (53, 54). Both J-chain and the IgA TP must be present with the correct Cys residues available for disulfide bond formation (6, 78–80), which may further require a disulfide-exchanging enzyme (53, 72). The presence of N-glycans on the TP, Cα3 domain, and J-chain and of other structural motifs in the IgA constant region are also critical for assembly formation and/or stability (13, 81). The intact assembly distribution could be specific to the nature of the antigen, strength of stimulation, exposure route, donor, and/or unique IgA1 clone (82). Secretion of dIgA1 could also depend on molecular chaperones like MZB1 or ERp44 (9, 55, 66, 67, 69, 70). Further investigation is critically important to understand what determines the extent of J-chain structural promiscuity of individual distinct IgA1 antibodies.

### Potential Implications of Structural Promiscuity on Human IgA Function.

We anticipate structural promiscuity could potentially have significant immunological consequences. Each molecule of a unique IgA clone recognizes the same antigen determined by the Fab variable region, irrespective of the antibody being in the mIgA or dIgA form. Given known differences between monomers and J-coupled dimers of IgA, however, two assembly states of the same unique clone could elicit very different immunological effects for the same target (18, 83, 84). For example, circulating mIgA immune complexes in particular are noted for inducing an immediate proinflammatory response by cross-linking FcαRI (CD89) (19, 85, 86). In contrast, in mucosal immune contexts, SIgA instead is known to contribute to homeostatic conditions by utilizing anti-inflammatory mechanisms such as immune exclusion and neutralization of pathogens. Here, dIgA is even able to intercept and neutralize invading viruses intracellularly during pIgR-mediated transepithelial transport to the mucosal lumen, which is inaccessible to mIgA due to the absence of J-chain (26, 27, 86, 87). Assays comparing mIgA1 and dIgA1 immunological functions thus represent key follow-up experiments.

Determining whether the structural promiscuity of individual IgA1 clones varies over time is also of high interest. If mIgA1/dIgA1 assembly ratios are dynamic, they could potentially reflect differences in immune response timing. Generation of high-affinity antigen-specific antibodies requires weeks, restricting initial responses to low-affinity antibodies. In these low-affinity responses, rapid production of exclusively J-coupled dIgA could compensate due to its enhanced avidity and neutralization capacity (27, 88, 89). Interestingly, antigen-specific circulatory IgAs produced early in response to viral or bacterial infection have indeed been described as predominantly polymeric and typically later replaced by a persistent monomer response (10, 17).

### Potential Implications for Shared Clonality between Serum and Mucosal IgA.

As shown here and in our earlier studies involving more donors (25, 38–40), clonal repertoires are entirely unique when comparing serum (or milk) samples from different donors (i.e., nearly every IgG1 or IgA1 Fab detected in each donor has its own unique mass/RPLC RT signature). However, we previously observed a high level of shared clonality between IgA1 from serum and breastmilk of the same donor (40), initially presumed to correspond solely to clones present in circulation as J-coupled dimers. From the structural promiscuity we have uncovered here, we now realize that the same distinct IgA1 found as SIgA at mucosal sites may not be exclusively dimeric in serum.

Adapting our approach to enable the comparison of assembly-specific IgA clonal repertoires in serum with those found in mucosal compartments is thus of high interest, as it should provide a clearer picture of the cellular origins, trafficking, clonality, and functions of the human IgA system (76, 90). Blood is the crossroad of not just circulatory Ig but also migrating plasmablasts and PCs, which may be actively secreting antibodies (91, 92). Notably, of these circulating ASCs, 80% are reportedly IgA+ (93). Several reports have also provided evidence suggesting stable, significant contributions to serum IgA by cells originally induced at mucosal sites (34, 63, 93–95).

Together this suggests each donor’s circulatory IgA clonal repertoire contains a broad, diverse mixture of antigen specificities, from all different inductive sites. We propose that J-chain structural promiscuity enables the structural and functional diversification of human IgA, exemplified by co-occurring clones. Too much clonal diversity risks autoimmunity, with negative health consequences especially in gut mucosal contexts home to both the largest population of IgA+ PCs and vast communities of microbes. By essentially copying the same IgA1 but changing the structural format, antibody diversity is increased while maintaining a restricted sequence repertoire. Considering the extensive, highly abundant distribution of human IgA throughout the body in many different compartments where diverse functions and signaling outcomes are required, this could thus, for example, enable serum IgA to function as a failsafe against sepsis if the mucosal epithelial barrier is breached (19, 96, 97).

### Outlook.

From a biotherapeutic development perspective, our findings could have significant impact. Nearly all antibody therapeutics have been designed using an IgG (mostly IgG1) template to date, and there is growing interest to develop alternatives based on the IgA class (98). With not only multiple N-glycans but also O-glycans present, IgA is one of the most heavily glycosylated human antibodies and structurally more complicated than IgG. However, the fact that IgA could be made into distinct modalities—i.e., mIgA and dIgA, with known differences in stability, clearance rates, and especially the higher avidity and enhanced neutralization capacity reported for J-coupled dimers—makes IgA-based therapeutics an exciting venue to explore (27, 99).

Taken together, our findings paint a broader, more complicated picture of human serum IgA in complete contrast with what is generally depicted in the literature (where the contributions of circulatory J-coupled IgA dimers are often ignored). This could result in overlooking nearly one-third of serum IgA, including many highly abundant unique clones, and potentially arriving at flawed or altogether-erroneous conclusions about the characteristics of human circulatory IgA if molecular and/or clonal composition is not directly assessed. The ability of the same distinct IgA1 antibody to occur both with and without the J-chain necessarily increases the complexity of serum IgA. This structural promiscuity of IgA with the J-chain introduces challenges, but more importantly it also inspires many exciting questions to address in the future to fully tease apart the intriguing system of human IgA.

## Materials and Methods

All donor samples were obtained with approval of the ethical advisory board and with written informed consent of the donors where applicable, as described in *SI Appendix*. A detailed description of all materials and methods used, including affinity purification, offline size exclusion chromatography (SEC), and generation of IgA1 Fabs, as well as for all MP and MS-based analyses, is provided in *SI Appendix*, *Supplementary Materials and Methods*.

Here in short, affinity purification of intact IgA assemblies from whole donor serum or plasma was achieved using CaptureSelect IgA Affinity Matrix which binds all subclasses/allotypes and all molecular forms of human IgA—including dimeric and secretory IgA—via the Fc domain. The molecular composition of circulatory IgA in these four donor samples was then investigated with single-molecule MP. With this label-free light scattering–based mass analysis technique, individual molecules are detected at the glass coverslip surface and the signal measured for each “event” is directly correlated to molecular mass. MP mass histograms depict the distribution of all detected molecules, allowing identification of each species by mass determined from Gaussian peak fitting and quantitative comparison of co-occurring molecular assembly populations. Constituent proteins, including IgA subclasses, were identified by MS-based label-free quantitative (LFQ) proteomics. For more details, see *SI Appendix*, *Supplementary Materials and Methods*.

For two donors in this study cohort, SEC was used to separate intact IgA assemblies directly in healthy donor plasma/serum for assembly-specific IgA1 Fab clonal profiling. This method was first optimized with SEC fractionation of intact IgA assemblies that had been affinity-purified from healthy human plasma/serum and of total plasma/serum, which were analyzed by MP and bottom–up LC–MS/MS proteomics to validate the separation. Following SEC separation of plasma/serum of Donors 2 and 4, identical quantities of two recombinant mIgA1 mAbs were spiked into each SEC fraction as internal standards. CaptureSelect IgA Affinity Matrix was then used to immobilize all IgA antibodies, followed by overnight digestion with an O-glycan-specific protease to specifically cleave the IgA1 hinge region. Such IgA1 Fabs prepared from individual SEC fractions containing either intact IgA monomer or J-coupled dimer following SEC separation of plasma/serum from Donors 2 and 4 were then subjected to intact LC–MS analysis. With this method a clonal profile containing the mass, RPLC RT, and abundance of every detected Fab is generated for each fraction which further directly indicates the intact assembly identity, allowing reconstruction of the intact IgA1 assembly SEC elution profile. For more details, see *SI Appendix*, *Supplementary Materials and Methods*.

## Supplementary Material

Appendix 01 (PDF)

## Data Availability

All data needed to evaluate the conclusions of this study are included in the article and/or *SI Appendix*. The raw RPLC-MS files and BioPharmaFinder intact deconvolution results from assembly-specific IgA1 Fab clonal profiling have been deposited to the MassIVE repository with the dataset identifier MSV000101556 (100).
